# Local In Vivo Measures of Muscle Lipid and Oxygen Consumption Change in Response to Combined Vitamin D Repletion and Aerobic Training in Older Adults

**DOI:** 10.3390/nu11040930

**Published:** 2019-04-25

**Authors:** D. Travis Thomas, David M. Schnell, Maja Redzic, Mingjun Zhao, Hideat Abraha, Danielle Jones, Howard Brim, Guoqiang Yu

**Affiliations:** 1 College of Health Sciences, University of Kentucky, Lexington, KY 40536, USA; 2 Department of Pharmacology & Nutritional Sciences, University of Kentucky, Lexington, KY 40536, USA; dave.schnell@uky.edu (D.M.S.); mre226@g.uky.edu (M.R.); hideat.abraha@gmail.com (H.A.); Danielle.Jones@uky.edu (D.J.); 3 Department of Biomedical Engineering, University of Kentucky, Lexington, KY 40536, USA; mingjun.zhao@uky.edu (M.Z.); howard.brim@uky.edu (H.B.); gyu2@uky.edu (G.Y.)

**Keywords:** skeletal muscle, IMCL, sarcopenia, metabolic function, vitamin D

## Abstract

Intramyocellular (IMCL), extramyocellular lipid (EMCL), and vitamin D deficiency are associated with muscle metabolic dysfunction. This study compared the change in [IMCL]:[EMCL] following the combined treatment of vitamin D and aerobic training (DAT) compared with vitamin D (D), aerobic training (AT), and control (CTL). Male and female subjects aged 60–80 years with a BMI ranging from 18.5–34.9 and vitamin D status of ≤32 ng/mL (25(OH)D) were recruited to randomized, prospective clinical trial double-blinded for supplement with a 2 × 2 factorial design. Cholecalciferol (Vitamin D_3_) (10,000 IU × 5 days/week) or placebo was provided for 13 weeks and treadmill aerobic training during week 13. Gastrocnemius IMCL and EMCL were measured with magnetic resonance spectroscopy (MRS) and MRI. Hybrid near-infrared diffuse correlation spectroscopy measured hemodynamics. Group differences in IMCL were observed when controlling for baseline IMCL (*p* = 0.049). DAT was the only group to reduce IMCL from baseline, while a mean increase was observed in all other groups combined (*p* = 0.008). IMCL reduction and the corresponding increase in rVO_2_ at study end (*p* = 0.011) were unique to DAT. Vitamin D, when combined with exercise, may potentiate the metabolic benefits of exercise by reducing IMCL and increasing tissue-level VO_2_ in healthy, older adults.

## 1. Introduction

Skeletal muscle metabolic dysfunction is the cornerstone of pathogenesis for most age-related co-morbidities. Understanding and combating muscle metabolic dysfunction in older adults has the potential to limit loss of strength and physical function [1], and improve quality of life. Vitamin D supplementation has been widely studied as a potential strategy to maintain physical function in aging [2,3,4] and vitamin D status has been positively associated with improving various metabolic outcomes [5,6,7,8]. While vitamin D insufficiency is estimated to contribute to $40 to $60 billion of economic burden annually [9], the mechanisms whereby vitamin D elicits physiological outcomes is not fully established in many tissues, and vitamin D interventions do not always result in improvements in physical function [10,11]. A better understanding of the effects of vitamin D deficiency and repletion, in relation to muscle metabolic function in aging is needed to further elucidate the metabolic benefits of vitamin D repletion in aging.

To date, exercise is the best known non-pharmacologic strategy to improve and maintain muscle function; however, response to exercise is diminished in the elderly, and the variability of response is increased. Several lines of current research indicate that vitamin D status may exacerbate this variability and skeletal muscle trophicity in general [12]. For example, low serum 25-hydroxyvitamin D (25(OH)D) is associated with increased muscle degeneration and fatty substitution [13], decreased muscle fiber size [14], and reduced muscle protein fractional synthetic rate [15]. Vitamin D deficiency has also been implicated in metabolic dysfunction associated with skeletal muscle including insulin resistance (IR) [16], inflammation [17], and extramyocellular lipid (EMCL) accrual [13,18]. Conversely, meta-analysis has shown that vitamin D supplementation increases muscle strength, with greatest improvements in those who have the most severe deficiency [19].

Recent reports suggest that vitamin D coupled with exercise has added positive effects on muscle mitochondrial function [6,7]. In addition, vitamin D receptor (VDR) expression in skeletal muscle is increased by both exercise [20] and vitamin D supplementation and has been associated with muscle regeneration and repair [14,21,22], suggesting an additive effect when exercise is combined with vitamin D repletion. Moreover, a synergistic interaction between exercise and vitamin D has been suggested [20]. Research in rodents has shown that exercise may improve skeletal muscle sensitivity to vitamin D by increasing the expression of CYP27B1 [20] and that vitamin D supports whole body β-oxidation [23], further supporting the connection between vitamin D and exercise [24]. Given these findings, it is conceivable that vitamin D deficiency coupled with aging and sedentary behavior [25] may contribute to muscle metabolic dysfunction. Furthermore, the age-associated decline in muscle metabolic function may be preceded by changes in intramuscular lipid [26]. Low levels of physical activity and vitamin D insufficiency appear to independently influence intramuscular lipid and may both contribute to impairments in muscle metabolic function [27,28]. This is important because derangements in muscle lipid metabolism are associated with mitochondrial dysfunction, muscle lipid stagnation, inefficient fat oxidation, and insulin signaling impairments [29]. Vitamin D has been inversely associated with EMCL [13,18], and to our knowledge, we were the first to report a positive linear relationship between vitamin D status and intramyocellular lipid (IMCL) in healthy, aged adults [27]. IMCL accumulation is linked to dysregulation of glucose homeostasis and IR in *sedentary* and *aged* individuals [30,31]. However, small amounts of moderate intensity aerobic exercise has been shown to increase oxidative capacity and overall fitness while expanding IMCL content [32,33]. Moreover, our IMCL findings [27] in a healthy, aged cohort were independent of body mass and daily physical activity, providing support to the notion of vitamin D dependent IMCL accretion. 

Coupled with these findings, along with evidence that short-term aerobic training (AT) promotes an increase in IMCL in aged adults [34], we hypothesized that vitamin D repletion promotes accumulation of IMCL; this IMCL is in turn more readily oxidized with the addition of AT. In order to test this hypothesis, the primary outcome of this study was to identify changes in muscle lipid ([IMCL]/[EMCL] ratio) in older adults following the combined treatment of vitamin D repletion and AT compared to vitamin D repletion alone, AT alone, and control conditions using magnetic resonance imaging (MRI) and spectroscopy (MRS). We also aimed to quantify the local muscle oxygen consumption rate (VO_2_) using near-infrared and diffuse correlation spectroscopies (NIRS/DCS) to evaluate how local muscle tissue metabolism is related to lipid partitioning between each treatment group.

## 2. Materials and Methods 

**Figure 1:**
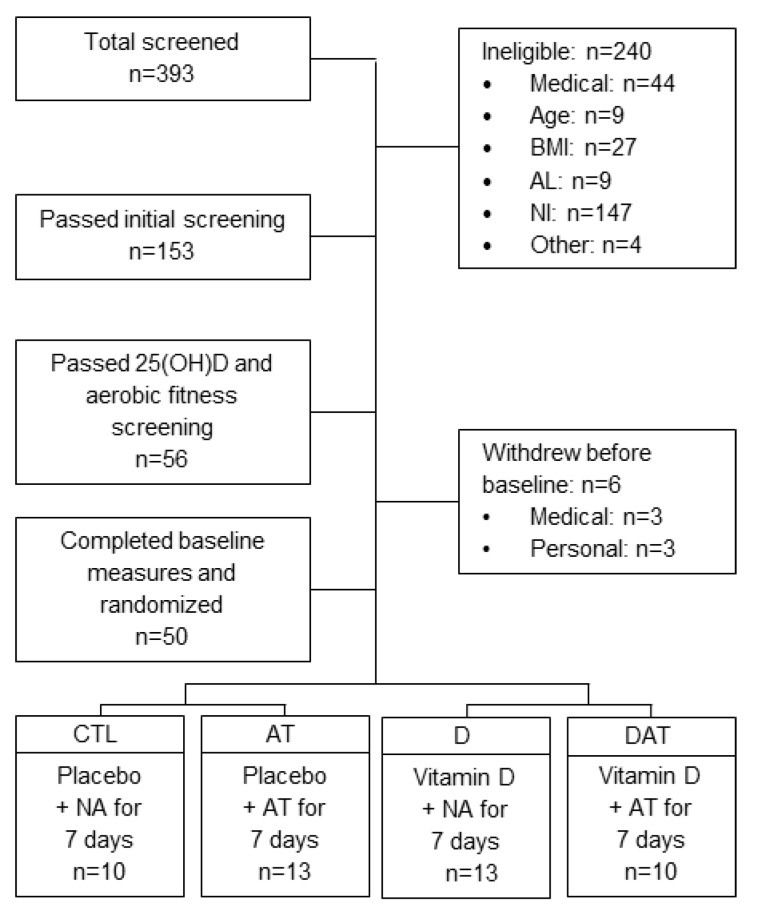
Recruitment for a randomized placebo-controlled clinical trial, in which aged adults completed seven consecutive days of aerobic training on a treadmill or continued usual activities (normal activity during the 13th week of vitamin D supplementation or place)bo. Serum 25(OH)D, serum 25-hydroxyvitamin D; AL, activity level; AT, aerobic training; CTL, control; D, vitamin D; DAT, vitamin D + aerobic training; NI, not interested; NA, normal activity.

Normal weight to class I obese (BMI = 18.5 to 34.9 kg/m^2^) subjects between the ages of 60 and 80 years of age were recruited. Subjects were not eligible if receiving treatment for vitamin D deficiency or if they had a recent history of vitamin D supplementation. Additional exclusion criteria included history of myopathy, neurologic disorders, disk disease, peripheral neuropathies, lower extremity surgery, or leg injury in the past 3 months. Final enrollment decisions were contingent upon serum 25(OH)D concentrations (≤ 32 ng/mL) and asymptomatic electrocardiogram findings from a graded treadmill exercise test (GXT). The study was approved by the university Institutional Review Board, and all eligible subjects gave informed written consent prior to engaging in baseline measures. All institutional and governmental regulations concerning the use of human volunteers were followed during this research. See Figure 1 and Table 1 for screening and accrual details and baseline characteristics. There were no adverse events associated with participating in this study. 

### 2.1. Study Design 

This was a randomized, prospective clinical trial double-blinded for supplement with a 2 × 2 factorial design (Clinical Trial Registry: NCT02221596). This 13-week trial was designed to compare the magnitude of change in the [IMCL]/[EMCL] ratio (primary outcome) and local muscle blood flow and oxygen consumption rate (secondary outcomes) following treatment with vitamin D repletion alone (D), aerobic training alone (AT), both vitamin D repletion and aerobic training (DAT), and control conditions (CTL). 

### 2.2. General Clinical Procedures

**Figure 2:**
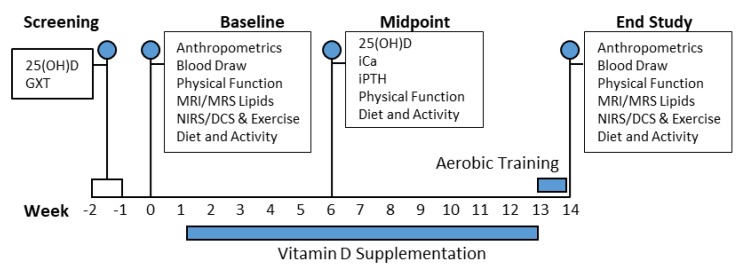
Schematic representation of the subject timeline. Serum 25(OH)D, serum 25-hydroxyvitamin D; GXT, graded treadmill exercise test; iCa, serum ionized calcium; iPTH, serum intact parathyroid hormone; MRI/MRS, magnetic resonance imaging/spectroscopy; NIRS/DCS, near-infrared spectroscopy/diffuse correlation spectroscopy.

Anthropometric assessments were completed at weeks 0 and 13 as indicated in the subject timeline (Figure 2). Blood draws were performed to measure serum ionized calcium (iCa) and intact parathyroid hormone (iPTH), vitamin D binding protein (VDBP), and 12-h fasting insulin during weeks 0, 6, and 13. Serum 25(OH)D, measured via LC–MS, was obtained during screening and was used as baseline values. VDBP, iPTH, and iCa variables were measured via ELISA to monitor the safety of our repletion strategy and provide a comprehensive systemic assessment of the response to our vitamin D supplementation intervention. Serum insulin was measured at baseline and endpoint to calculate HOMA-IR and to subsequently assess insulin secretion and insulin sensitivity. The coefficient of variation was < 10% for all blood analyses. MRI and MRS measures of gastrocnemius EMCL and IMCL were collected on the same testing day as anthropometrics measures (weeks 0 and 13). Following MRI, we collected NIRS/DCS optical data in the same region of the gastrocnemius. We integrated NIRS/DCS technology with our gastrocnemius plantar flexion fatigue protocol (described below) to assess changes in local skeletal muscle metabolic function from baseline to endpoint. Following baseline testing, all subjects were randomized to either DAT, D, AT or CTL treatment.

Subjects were instructed to maintain their usual dietary patterns throughout the duration of the study and energy intake was assessed via 24-hr dietary recall. Dietary recalls at each study time point consisted of 3 randomly selected, non-consecutive days including 1 weekend and 2 week days for a total of 9 recalls. Energy content was determined using Nutrition Data System for Research (NDS-R, Version 2017, University of Minnesota, Minneapolis, MN, USA). 

Subjects wore an ActiGraph (MTI, Inc. Fort Walton Beach, FL, USA) to measure average daily activity over a 7-day data collection period during each time point. A complete description of the ActiGraph and its technical reliability have been published elsewhere [35,36]. The data collection interval was set at one minute with a minimum wear of 12 h required to constitute a valid day. 

During screening, all subjects completed a graded treadmill exercise test (GXT) using a VMAX Encore metabolic cart (Vyaire, Yorba Linda, CA, USA) with O_2_ measures to derive systemic VO_2max_. This multi-staged treadmill GXT was performed using progressive 2-min workload stages. The initial speed of the treadmill was set based on the participant’s reported fitness level prior to the start of the test in an attempt to limit the test time to between 8–12 min in accordance with the American College of Sports Medicine (ACSM) Guidelines for Exercise Testing and Prescription [37]. 

### 2.3. Vitamin D and Placebo Supplementation Protocol

Vitamin D repletion occurred in a double-blinded randomized placebo-controlled fashion. Vitamin D repletion goals were set to increases 25(OH)D from 20–32 ng/mL to greater than 40 ng/mL. We chose this repletion target because it is a commonly referenced concentration hypothesized to maintain extra-skeletal health [38]. Our repletion strategy was designed to promote a 25(OH)D steady state [39], correct vitamin D insufficiency, and was similar with previous dosing strategy that reported changes in muscle metabolic function [6,39]. The study coordinator was blinded to supplement assignment and provided supplement allocations every 2 weeks. The study coordinator instructed subjects to self-administer 2 gelcaps at the same time by mouth daily during the morning hours (10,000 IU/day × 5 days/wk Cholecalciferol (vitamin D_3_) or placebo). Supplement compliance was determined by subject self-reports and pill counts. Any unused gelcaps were returned to study personnel at week 6 and at endpoint. 

BioTech Pharmacal, Inc. supplied all vitamin D and placebo gelcaps and has supplied vitamin D and placebo for previously published clinical trials. The company follows FDA GMP standards for quality assurance for physical attributes, capsule content, and purity. Vitamin D and placebo gelcaps were identical in size, weight and appearance. Vitamin D gelcaps were encapsulated in a clear gelatin two-piece capsule (size 4), filled with white powder and the active ingredients, vitamin D_3_ (Cholecalciferol) 5000 IU with microcrystalline cellulose, and gelatin (inactive ingredients). Placebo capsules were identical and filled with microcrystalline cellulose, gelatin (inactive ingredients) and no active vitamin D. Both products met USP specifications for capsule weight variation and met USP and Prop. 65 guidelines for heavy metals.

### 2.4. Aerobic Training Protocol

Our exercise intervention was based on an acute 7-day intervention consisting of daily aerobic exercise [34] in older adults that resulted in intramuscular lipid repartitioning. For seven consecutive days during week 13, subjects randomized to AT or DAT performed 60 min of treadmill exercise at an intensity up to 60–65% of their maximum heart rate (approximately 55–65% of their maximal baseline aerobic capacity). During exercise sessions, subjects wore heart rate monitors (Polar T31; Polar Electro, Kempele, Finland) to monitor target heart rate. 

### 2.5. Muscle Lipid Assessment

Lipid was quantified using single voxel, hydrogen magnetic resonance spectroscopy (MRS) to distinguish IMCL from EMCL. Subjects were positioned in supine orientation with their lower leg in the transmit/receive knee coil in a fixed dorsiflexed position parallel to the main magnetic field, a 3.0T TIM TRIO scanner (Siemens, Erlangen, Germany). Spectral fitting was carried out using the software package LcModel (Stephen Provencher Inc., Oakville, ON, Canada). Then, 1H-MRS was used to quantify IMCL (TR/TE = 2000/30 ms). EMCL was quantified using T1 scans with and without fat saturation and an automated segmentation program, *FMRIB* Software Library version 5.0 (FMRIB, Oxford, UK). Sets of T1-weighted images (TR/TE = 650/22 ms) for high-resolution anatomy were used to assess EMCL. Medial and lateral heads of the gastrocnemius were quantified and combined to assess total EMCL content. EMCL was reported as percent of gastrocnemius volume (%_gast_), and IMCL was reported as the ratio of IMCL:water spectral abundance ×1000 (AU).

### 2.6. Fatigue Gastrocnemius Plantar Flexion Protocol Coupled with Hybrid Diffuse Optical Spectroscopy (NIRS/DCS)

At baseline and endpoint, subjects were seated at 70° in a semi-recumbent position on a dynamometer (BTE Primus RS, Hanover, MD, USA) with the calf exposed for easy placement of optical spectroscopy probes. Following a warm up, a minimum of five maximal voluntary isometric plantar contractions (MVC) were completed to determine maximum torque generation. MVC attempts were repeated until coefficient of variance fell below 10%, and the highest concentric plantar-flexion peak-torque value was recorded as the MVC. A 3-min rest was provided prior to the start of fatigue exercise to allow for pre-fatigue NIRS/DCS measures. Following MVC determination, an optical probe connected to the hybrid diffuse optical instrument (NIRS/DCS) combining a commercial NIRS oximeter (Imagent, ISS Inc., Champaign, IL, USA) and a custom built DCS flowmeter was placed over the right calf (medial gastrocnemius) and attached to the belly of the muscle. Gastrocnemius hemodynamics, including blood flow (BF), blood oxygen saturation (StO_2_), and oxygen consumption rate (VO_2_), were continuously measured from 3 min prior to the start of the fatigue exercise to 15 min after its completion [40,41]. Subjects were asked to complete 75 repetitions of isotonic concentric plantar-flexion contractions at maximal voluntary angular velocity with torque set to 35% of MVC. Optical hemodynamic data were collected at the end of every concentric phase plantar-flexion (0.5 s measurement periods) during exercise to reduce motion artifact [40,42]. Optical measurements were normalized to a resting optical baseline before baseline and endpoint measurements; relative BF (rBF) and relative VO_2_ (rVO_2_) were then calculated by methods described elsewhere [41]. Change in StO_2_ (ΔStO_2_) was calculated as the increase in StO_2_ at all measurement times from resting StO_2_ to minimize the influence of individual variation in resting StO_2_ and to enhance the StO_2_ response induced by the fatigue exercise. Post-fatigue exercise recovery in rVO_2_ was characterized by the recovery half-time (T50), a time interval from the end of exercise to the time that they reached a half-maximal value. 

### 2.7. Statistical Methods

Sample size was estimated based on the expectation that AT groups would have a lower [IMCL]/[EMCL] ratio, with the lowest ratio in DAT. Previous research measuring EMCL and IMCL in vivo reported changes of −5.8 ± 3.4%_gast_ and 0.5 ± 0.2 AU, respectively, following a 7-day diet and exercise intervention [34]. With the assumption that vitamin D combined with AT change EMCL and the [IMCL]/[EMCL] ratio changes similarly and that the change is smallest in the group without vitamin D or AT, a two-way analysis of variance (repeated measures (RMANOVA)) was estimated to have at least 80% power to detect the main effects of vitamin D and AT (sample size is 11 per group, *n* = 44). IMCL and EMCL comparisons between groups were also assessed using two-way RMANOVA to align with our primary outcome analysis. We suspected that small differences in baseline IMCL could contribute directly to the determination of the size of the differences at endpoint. For this reason, baseline IMCL values were included as a covariate and were included in secondary analysis of intervention effects using Analysis of Covariance (ANCOVA).

RMANOVA was also used to compare NIRS/DCS outcomes in all study groups for secondary outcomes. Fisher’s least significant difference (LSD) was used for post hoc analysis to identify specific group differences when main effect ANOVA *p* < 0.05. Exploratory analysis included examining correlation coefficients using Pearson’s *r* between 25(OH)D and iPTH, Δ25(OH)D and ΔIMCL, and other physiological variables. Within group comparisons of muscle lipid depots were exploratory and assessed using paired t-test between baseline and endpoint. All analyses were performed using SPSS^®^ statistical software version 22.0 (IBM Corporation, Armonk, NY, USA) and significance was defined as *p* < 0.05. Values presented as mean ± SEM.

## 3. Results

### 3.1. Muscle Lipid

For our primary outcome of examining differences in the [IMCL]/[EMCL] ratio, there were no significant effects in response to treatment (supplement, *p* = 0.121; treadmill, *p* = 0.178; interaction, *p* = 0.915) and no time or group main effects (*p* = 0.29). When analyzed separately, there were no significant differences in IMCL or EMCL by time and/or supplement or treadmill training (Table 2). However, a significant difference in mean IMCL change (*p* = 0.049; η^2^ = 0.301) between intervention groups was observed when controlling for baseline IMCL variability using ANCOVA. A trend in DAT IMCL reduction was observed (Table 2), while a mean increase (1.11 ± 0.32 AU) was observed in all other groups combined (*p* = 0.008). There were no sex differences in the [IMCL]/[EMCL] ratio, IMCL, or EMCL by time and/or by group.

**Figure 3:**
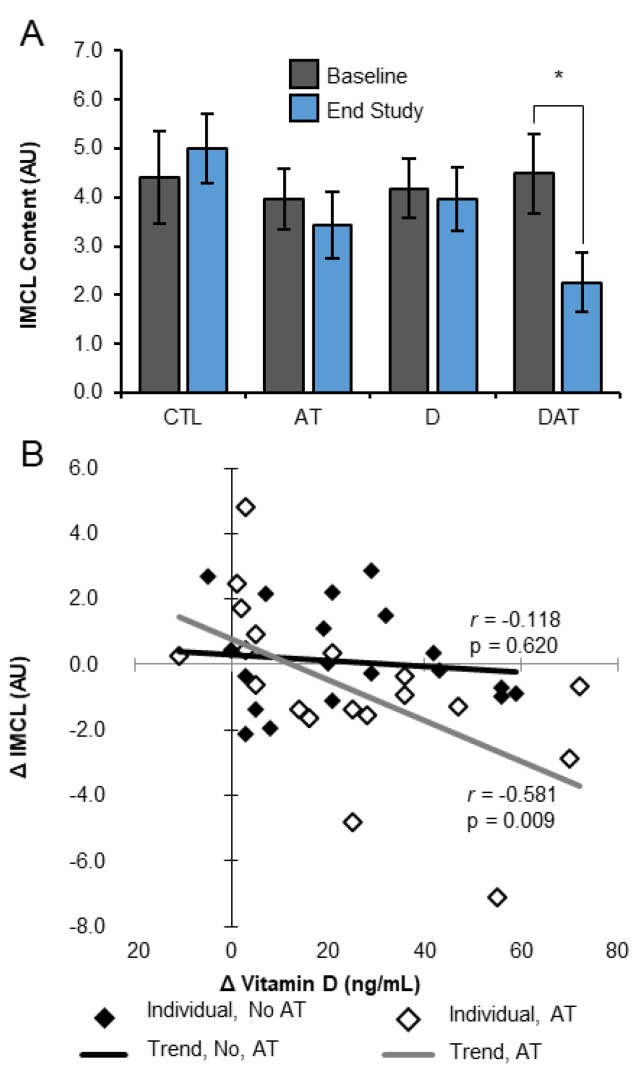
IMCL response to 13 weeks of intervention. (**A**) DAT decreases IMCL from baseline after 13 weeks. Bars represent mean ± SEM; * *p* < 0.05; paired t-test. (**B**) Change in IMCL content proportional to change in 25(OH)D concentration from baseline to endpoint. Serum 25(OH)D, serum 25-hydroxyvitamin D; AT, aerobic training; AU, arbitrary units; CTL, control; D, vitamin D; DAT, vitamin D + aerobic training; IMCL, intramyocellular lipid.

Secondary, exploratory analysis using paired sample t-tests revealed a significant within group change in IMCL in DAT only (*p* = 0.035) (Figure 3A). The change in IMCL was inversely correlated with change in 25(OH)D in AT and DAT (*r* = −0.581, *p* = 0.009), but not in sedentary D and CTL counterparts (*r* = −0.118, *p* = 0.620) (Figure 3B). Within group comparisons did not reveal significant changes in the [IMCL]/[EMCL] ratio or EMCL. 

### 3.2. Fatigue Test with Local Muscle Tissue BF, StO_2_ and VO_2_

There were no differences between intervention groups in baseline MVC (Table 1). NIRS/DCS measures for both baseline and endpoint were acquired for 37 subjects. Demographic variables (Table 1) did not correlate with hemodynamic NIRS/DCS data at baseline. 

**Figure 4:**
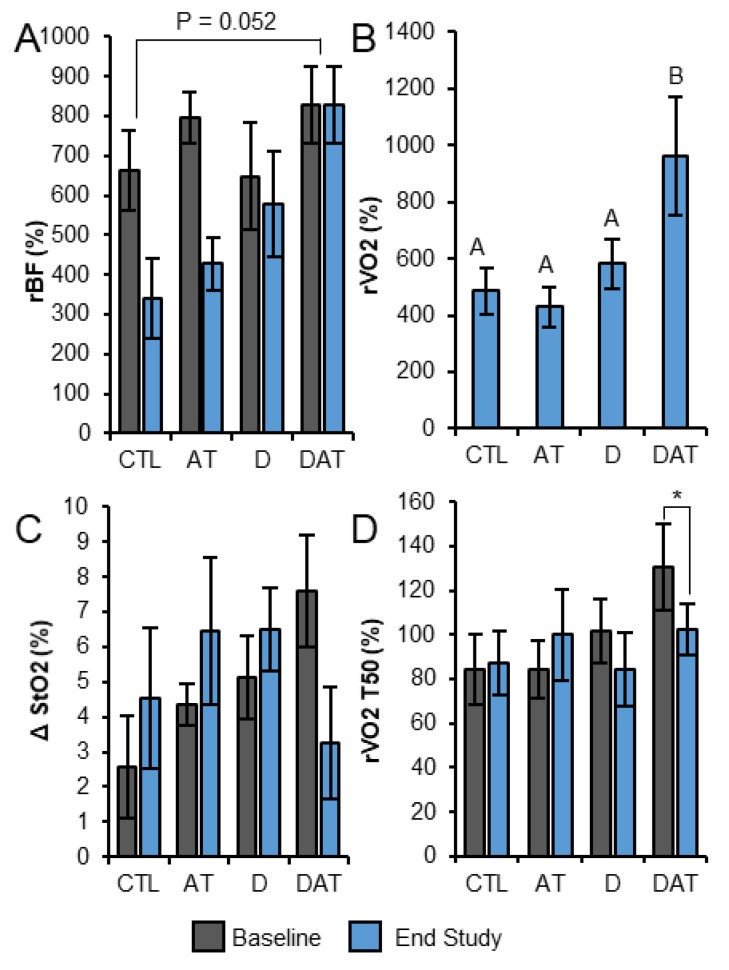
NIRS/DCS measurements in response to 13 weeks of intervention. (**A**) Relative blood flow during exercise by group. (**B**) Oxygen consumption by group during exercise at study endpoint. (**C**) Change in Oxygen saturation by group at study baseline and endpoint at 8 min of recovery after plantar flexion exercise. (**D**) Recovery half-time of oxygen consumption at study baseline and endpoint. Bars represent mean ± SEM; labeled means without a common letter differ, *p* < 0.05, ANOVA (A) RMANOVA (B); * *p* < 0.05, paired *t*-test. AT, aerobic training; CTL, control; D, vitamin D; DAT, vitamin D + aerobic training; rBF, relative blood flow; ΔStO_2_, pre- to post-exercise change in oxygen saturation; rVO_2_, relative oxygen consumption; rVO_2_ T50, relative oxygen consumption recovery half-time.

**Figure A1:**
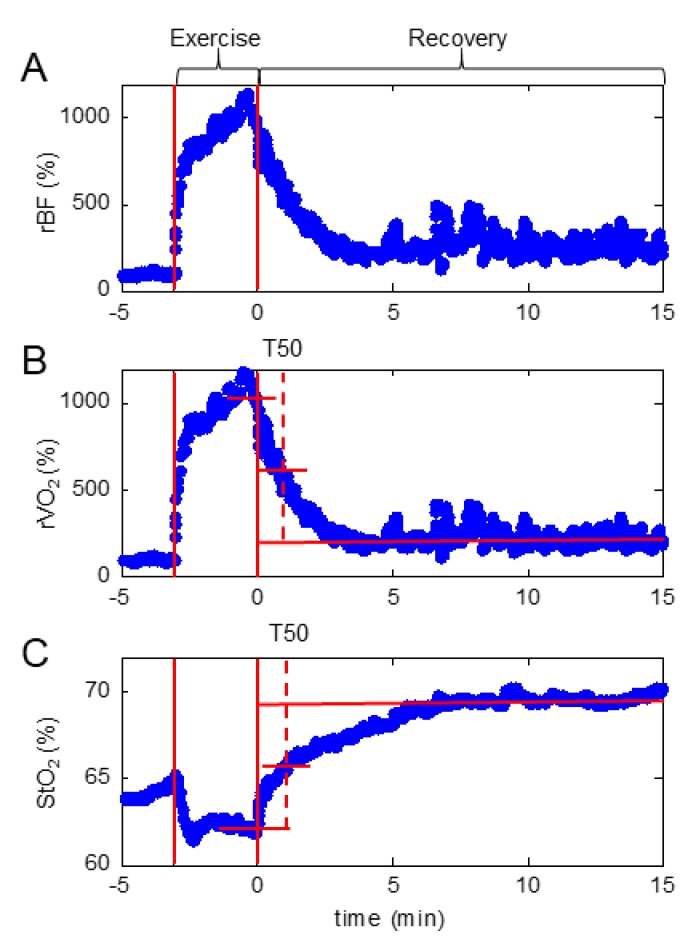
Representative profiles of a typical response seen in subjects when completing the fatiguing exercise. Standard illustrative profile of (**A**) relative blood flow, (**B**) relative oxygen consumption rate, and (**C**) blood oxygen saturation in a subjects throughout fatiguing exercise. Solid vertical lines indicate the beginning and the end of fatiguing exercise. Horizontal lines indicate end of exercise, post-exercise steady-state, and midpoint values. Hashed vertical lines illustrate the recovery half-time (T50) of relative oxygen consumption following fatiguing exercise. rBF, relative blood flow; rVO_2_, relative oxygen consumption; StO2, oxygen saturation.

Figure A1 illustrates a typical hemodynamic response in rBF, rVO_2_, and StO_2_ in human subjects during fatiguing exercise. Our secondary outcome analysis of NIRS/DCS data during the fatiguing exercise protocol indicated a significant effect of supplementation (*p* = 0.017, η^2^ = 0.140) in rBF. Further analysis using ANOVA revealed a main effect (*p* = 0.049), and post hoc analyses indicated a strong trend towards rBF increase from CTL to DAT (*p* = 0.052) (Figure 4A). At baseline there were no group differences in blood flow during exercise. DAT endpoint rBF (827 ± 488 percent baseline) was higher than CTL (385 ± 148 percent baseline, *p* = 0.010) and AT (426 ± 332 percent baseline, *p* = 0.013). There were no significant group by time interactions in rVO_2_ during exercise; however, secondary analysis by ANOVA revealed DAT was significantly higher at endpoint than CTL (*p* = 0.007), AT (*p* = 0.002), and D (*p* = 0.013) (Figure 4B). No significant differences in ΔStO_2_ were found between intervention groups during fatiguing exercise at baseline or at the end of the study. 

At endpoint, rBF, rVO_2_, and StO_2_ progressed towards pre-fatigue exercise values during the immediate recovery period (min 1 to 3) following fatiguing exercise. Throughout 15 min of recovery from the fatiguing exercise, there were no main effect differences in rBF, rVO_2_, or StO_2_ by time or by group. We examined shifts in ΔStO_2_ from baseline to endpoint for each minute of recovery (min 1–15) and found a significant time by supplement by treadmill interaction effect in ΔStO_2_ from 2 min onward (*p* = 0.027, η^2^ = 0.125). This difference reached its maximum at 5 min of recovery and maintained a steady state from 8–15 min (Figure 4C). 

While there were no intervention differences by time and/or by group (supplement *p* = 0.138; treadmill training *p* = 0.957) in rVO_2_ T50, paired t-tests showed a significant decrease in rVO_2_ T50 for DAT (−28.7 ± 31.3 s, *p* = 0.030) (Figure 4D). There were no differences among treatments in StO_2_ T50. There were no differences between sexes for any NIRS/DCS variables.

### 3.3. Other Outcomes

For D and DAT, mean 25(OH)D concentrations increased over time to 54.1 ± 10.8 and 63.7 ± 17.7 ng/mL at midpoint and endpoint in D and to 62.0 ± 14.3 and 72.1 ± 16.3 ng/mL at midpoint and endpoint in DAT, respectively (*p* < 0.001) (Table A1). Change in 25(OH)D was not affected by treadmill training (*p* = 0.387). Serum 25(OH)D was 31.2 ± 9.7 and 32.6 ± 10.6 ng/mL at midpoint and endpoint for AT and 29.5 ± 10.2 and 31 ± 10.4 ng/mL at midpoint and endpoint in CTL. Serum 25(OH)D changes in AT and CTL were also significant by time (*p* < 0.001), and remained significantly lower when compared to D (*p* < 0.001) and DAT (*p* < 0.001) (time x group post hoc comparisons). Intact PTH significantly changed over time, and changes in 25(OH)D concentrations were negatively associated with iPTH (*r* = −0.486, *p* = 0.001) in the total sample. This appeared to be driven by DAT (*r* = −0.725, *p* = 0.027). Average subject-reported compliance with vitamin D and placebo supplementation was 93% and 96%, respectively. 

HOMA-IR, VDBP and iCa were not significantly different between groups at any study time point. Physical activity, total caloric intake, dietary fat and vitamin D intake were not significantly different between intervention groups at baseline or over time (See Table A1).

## 4. Discussion

This was the first clinical trial utilizing novel non-invasive techniques, to examine the combination of vitamin D repletion and AT on muscle lipid and metabolic function in aged muscle. Although our primary analysis examining change in the ratio of intramyocellular lipid (IMCL) to extramyocellular lipid (EMCL) was not significant, our secondary analysis of IMCL, secondary outcomes and exploratory analyses suggested that vitamin D repletion augments aerobic exercise induced IMCL reduction and promotes improvements in muscle metabolic function. Specifically, the combination of aggressive vitamin D repletion and short-term aerobic training (AT) produce the greatest loss of IMCL with concomitant increases in local muscle tissue relative oxygen consumption (rVO_2_). Combination treatment also produced other positive hemodynamic changes including increased relative blood flow (rBF) and rVO_2_ as well as decreased change in oxygen saturation (ΔStO_2_) and rVO_2_ recovery half time (T50). All of these changes are consistent with improved muscle metabolic function and exercise recovery potential. 

These findings were observed in a healthy cohort of aged, normal weight, insulin-sensitive, community dwelling subjects and were in partial support of a two-part study hypothesis. First, these findings do not support the first half of our hypothesis, which stated that vitamin D repletion promotes accumulation of IMCL in aged human subjects. Our previous work reported a positive linear relationship between 25-hydroxyvitamin D (25(OH)D) and gastrocnemius IMCL in a cross-sectional sample of healthy, aged adults, independent of physical activity and body mass [27]. The D group in the present study did not experience an increase in IMCL content over 13 weeks, despite increasing mean 25(OH)D concentration from 26 to 64 ng/mL. While our current findings do not support our previous work in humans, other work from our group [5,43] shows that vitamin D is involved in lipid droplet filling and gene expression of the adipose related protein, perilipin 2 (PLIN2). We acknowledge that the “quality” of IMCL and factors including lipid species distribution, lipid droplet size, and subcellular localization of lipid droplets may be associated with the capacity to turnover lipid efficiently and may be more important than absolute IMCL content [5,44,45,46]. Nevertheless, one explanation for not seeing a significant IMCL increase in the D group might be that the time spent participating in moderate physical activity as we measured by Actigraph acceleometry was maintained at approximately 1 h per day throughout the intervention and may have provided significant lipolytic stimulus to maximize lipid turnover and minimize myocellular lipid filling [44]. 

The second half of our hypothesis—that IMCL is more readily oxidized with the addition of AT—was supported by our current data suggesting that IMCL is more readily used by skeletal muscle with the addition of aerobic training. Data from DAT suggests that IMCL is more readily oxidized in the presence of vitamin D sufficiency. Significant change in IMCL storage and muscle metabolic function (∆StO2, rVO_2_, rVO_2_ T50) were only observed after vitamin D repletion followed by aerobic training with no appreciable changes seen in CTL or subjects who only received aerobic training or vitamin D repletion. This is significant because augmenting IMCL utilization during AT may serve to reduce muscle metabolic dysfunction in aging [44] and help to alleviate progressive loss of strength and function over time.

The loss of IMCL experienced by DAT could be explained by several mechanisms including improved blood flow and improved mitochondrial function. Previous studies suggest that vitamin D coupled with exercise has added positive effects on muscle mitochondrial function [6,7]. In addition, vitamin D receptor (VDR) expression in skeletal muscle was increased by both exercise [20] and vitamin D supplementation and has been associated with muscle regeneration and repair [14,21,22]. These independent effects may suggest an additive effect when exercise is combined with vitamin D repletion. However, the mechanistic link between vitamin D repletion and exercise, muscle lipid management, oxidation, and muscle metabolic function in aging, requires further study. Although vitamin D has been linked with increased PLIN2 expression and lipid filling, others have suggested that increased PLIN2 availability on lipid droplets may help increase rates of β-oxidation [46]. Furthermore, several lipid metabolism pathways involving diacylglycerol acyltransferase (DGAT), peroxisome proliferator-activated receptor α (PPARα) and its target gene carnitine palmitoyltransferase-1 (CPT-1) have been positively associated with vitamin D [21,47] but require further investigation in aging.

The lack of change in EMCL was likely due to the short duration of the exercise protocol, lack of significant body weight maintenance, and the large size of the EMCL lipid depot that is less sensitive to change, particularly when body mass remains stable. Although EMCL appears to be inversely related to vitamin D status in other published studies [13,18], short-term aerobic exercise and vitamin D repletion had no effect in this study.

Our key secondary outcome findings were that rVO_2_ was significantly higher in DAT during the endpoint fatigue protocol and that these results matched the significant loss of IMCL in the same group of subjects. These observations not only show that our 13-week vitamin D repletion + AT protocol promotes the greatest IMCL loss, but also suggests from our non-invasive NIRS/DCS optical findings that IMCL is more readily oxidized with the addition of aerobic exercise following vitamin D repletion. 

These findings also align with previous work showing that vitamin D is associated with improvements in oxygen consumption in vitro [7] and skeletal mitochondrial capacity during plantar flexion exercise in clinical setting [6]. Moreover, our group has recently shown that vitamin D can elicit many other distinct effects on myocellular lipid partitioning and lipid packaging potential. This includes changing IMCL subspecies content, partitioning of intramuscular triglycerides between myotubes and myoblasts, and increasing expression of genes involved in lipid droplet packaging and lipolysis [5]. 

The DAT intervention also had a significant effect on measures of rBF, ΔStO_2_, and recovery half-time oxygen consumption rate (rVO_2_ T50). To our surprise, group comparisons of rBF during fatiguing exercise from study baseline to endpoint indicated a trend towards an increase in D (compared to CTL and AT) at endpoint, while DAT rBF was significantly higher than CTL despite no group differences in these variables observed during baseline assessment. These observations deserve further attention in future studies given the potential impact of an additive or synergistic effect of a vitamin D repletion and exercise intervention could have on maintaining muscle metabolic health. A connection between vitamin D and factors related to blood flow such as flow-mediated dilation, vascular endothelial function, endothelial nitric oxide synthase, and erythropoiesis have been documented [48,49,50], but the specific relationship between exercise and vitamin D on blood flow outcomes is not well described [48,51]. 

Finally, we examined shifts in ΔStO_2_ from study baseline to endpoint for each minute of recovery (min 1–15) to better represent StO_2_ response to our acute fatigue protocol. DAT was the only group to significantly change oxygen saturation towards full recovery from study baseline to endpoint and DAT ΔStO_2_ was significantly reduced from minute 2 through minute 15. A reduction in ΔStO_2_ indicates enriched mitochondrial capacity by increasing oxygen extraction to muscles [52]. While it appears that DAT is increased at baseline relative to other groups, this difference was not statistically significant, approaching significance most closely at 2 min (*p* = 0.231, ANOVA). Finally, both vitamin D-supplemented intervention groups (D and DAT) experienced a declining trend decline in their recovery half time (rVO_2_ T50), but only DAT experienced a significant decline over time (pre- to post intervention). We interpret a reduction in DAT rVO_2_ T50 as a beneficial alteration and potentially an adaptation to support increased muscle oxygen utilization, suggesting improved mitochondrial function [52,53]. Together, these changes in hemodynamics and oxygen consumption may be attributed to multiple mechanisms including improved muscle blood flow and oxygen delivery creating a reduction in oxygen debt, [54] improved lipid substrate availability to the mitochondria [32,55], and direct improvements of mitochondrial function [56,57]. 

In summary, near-infrared spectroscopy/diffuse correlation spectroscopy (NIRS/DCS) data show increased relative oxygen consumption rate and relative blood flow to active muscles and faster exercise recovery in subjects receiving DAT. This suggests that combining aerobic exercise with vitamin D repletion may help improve muscle metabolic function by influencing local muscle tissue aerobic capacity and post exercise recovery. To our knowledge, these are the first data to show the relationship between vitamin D repletion, aerobic exercise, and muscle metabolic function with the combined use of inexpensive and non-invasive NIRS/DCS with magnetic resonance spectroscopy (MRS). 

The strengths of this study include a randomized design, participant commitment (≥ 95% compliance to AT program and ≥ 93% adherence to supplementation), low study dropout rate, and the blinding of subjects, exercise trainers and study team. Other strengths involved the inclusion of both sexes, monitoring physical activity, dietary energy intake, and knowledge of each subject’s insulin sensitivity. 

There are also limitations to consider when interpreting these results. While adequately powered when designed, variability in IMCL data was higher than expected and limited our ability to observe significant group differences when analyzing our primary outcome with RMANOVA. Lost data also contributed to study limitations since pre-post IMCL data could only be analyzed in 40 of the 50 subjects recruited. Lost IMCL data were explained by, missing data, uninterpretable MRS spectra caused by low signal:noise, and logistical/scheduling complications. Nine subjects did not have optical spectroscopy data due to either NIRS/DCS or BTE machine instrument failure (*n* = 7 [CTL *n* = 1; AT *n* = 2; D *n* = 1; DAT *n* = 3]) or unreliable measurements due to noise (*n* = 2 [CTL *n* = 1; DAT *n* = 1]). Furthermore, the study sample was comprised of 93% white subjects, limiting our ability to translate findings to other racial and ethnic groups. Vitamin D binding protein (VDBP) genetic polymorphisms are associated with race and could alter VDBP concentration and binding affinity [58,59,60]. The functional significance of these genetic polymorphisms is not completely understood, but VDBP can influence vitamin D status and should be considered in future studies. Moreover, our stringent eligibility criteria set forth in an effort to establish and maximize a controlled scientific model of study and establish proof of concept also limits our ability to generalize our results to all aged adults. Finally, we acknowledge that our muscle lipid and hemodynamic findings do not provide direct evidence of IMCL turnover and improved mitochondrial function. However, this work provides novel non-invasive insight to inform future mechanistic studies and future translational work to fully understand the mechanistic underpinnings outlining the benefit of combining exercise and vitamin D treatment on skeletal muscle metabolism. 

## 5. Conclusions

In conclusion, the [IMCL]/[EMCL] ratio did not change in our sample of healthy aged subjects. However, these results partially supported our hypothesis that IMCL reduction would be enhanced with the addition of aerobic training following vitamin D repletion, but vitamin D repletion alone did not increase IMCL over 13 weeks. Vitamin D repletion, when combined with AT, may potentiate the metabolic benefits of exercise in aging by reducing IMCL and altering skeletal muscle tissue-level VO_2_. Future work should test the hypothesis that vitamin D promotes muscle lipid availability for β-oxidation in response to exercise, thereby preventing lipotoxicity and improving muscle anabolic sensitivity in populations at risk for sarcopenia and cachexia.

## Figures and Tables

**Table 1:** Baseline characteristics of the 46 subjects who completed 13 weeks of supplementation with vitamin D or placebo with the 13th week consisting of AT or usual physical activities.

Category	Measurement	CTL	AT	D	DAT
Demographics	*N* (Male/Female)	10 (3/7)	13 (7/6)	13 (6/7)	10 (5/5)
	Age, years	67.5 ± 2.1	68.6 ± 1.3	65.9 ± 1.6	67.9 ± 1.9
	White	9	13	12	9
	Asian	1	0	1	0
	African American	0	0	0	1
Anthropometric Measurements	Height, cm	167 ± 3.0	170 ± 2.0	168 ± 3.0	169 ± 3.0
	Weight, kg	75.1 ± 3.8	72.2 ± 3.4	76.1 ± 4.1	74.6 ± 5.0
	BMI, kg/m	27.0 ± 1.2	24.7 ± 0.8	26.6 ± 0.9	26.2 ± 1.5
Serum Measurements	25(OH)D, ng/mL	24.8 ± 2.1	25.2 ± 1.4	26.6 ± 1.2	27.1 ± 1.6
	VDBP, µg/mL	110 ± 5	114 ± 3	111 ± 4	115 ± 5
	iPTH, pg/mL	36.8 ± 3.6 ^AB^	32.4 ± 2.6 ^B^	53.1 ± 5.5 ^C^	47.6 ± 5.0 ^BC^
Physiological Measurements	VO_2_max, mL/(kg min)	25.8 ± 1.8	28.0 ± 2.9	28.4 ± 2.1	27.0 ± 2.8
	Light PA, min/d	878 ± 67	839 ± 53	956 ± 68	806 ± 32
	Moderate PA, min/d	63.2 ± 11.7	52.0 ± 5.9	59.4 ± 7.7	69.6 ± 11.1
	Vigorous PA, min/d	0.28 ± 0.14	0.21 ± 0.11	0.69 ± 0.69	1.15 ± 0.82
	MVC, *N* m	128 ± 7.6	139 ± 10.9	149 ± 8.5	140 ± 10.7
	Energy Intake, kcal/d	1741 ± 156	2045 ± 325	1457 ± 119	1789 ± 241

All values are mean ± SEM. Serum 25(OH)D, serum 25-hydroxyvitamin D; AT, aerobic training only; CTL, control; D, vitamin D only; DAT, vitamin D + aerobic training; MVC, maximum voluntary contraction; iPTH, intact parathyroid hormone, PA, physical activity; VDBP, vitamin D binding protein; VO_2_max, maximal aerobic capacity. Values not sharing a letter are significantly different (*p* < 0.05, ANOVA, Fishers LSD).

**Table 2:** Gastrocnemius muscle lipid before and after 13 weeks of supplementation with vitamin D or placebo with the 13th week consisting of AT or usual physical activities.

Value	Time point	CTL	AT	D	DAT
IMCL	Baseline	4.84 ± 0.94	3.93 ± 0.67	3.93 ± 0.61	4.27 ± 0.93
	Endpoint	5.01 ± 0.72	4.10 ± 0.92	3.95 ± 0.65	2.26 ± 0.61
	Change	0.16 ± 0.54	0.17 ± 0.68	0.02 ± 0.47	−2.02 ± 0.78 *
EMCL	Baseline	27.3 ± 2.4	26.0 ± 1.1	28.0 ± 1.8	25.8 ± 1.4
	Endpoint	25.5 ± 2.0	25.7 ± 1.4	28.0 ± 1.5	25.2 ± 1.8
	Change	−1.7 ± 1.4	−0.3 ± 1.0	−0.4 ± 1.0	−0.6 ± 0.7
IMCL:EMCL	Baseline	0.162 ± 0.034	0.156 ± 0.025	0.150 ± 0.022	0.183 ± 0.039
	Endpoint	0.218 ± 0.041	0.164 ± 0.040	0.141 ± 0.022	0.138 ± 0.048
	Change	0.056 ± 0.045	0.008 ± 0.028	−0.001 ± 0.018	−0.041 ± 0.040

Intramyocellular lipid (IMCL) was calculated by magnetic resonance spectral abundance relative to total water content. Extramyocellular lipid (EMCL) was calculated as percent of medial and lateral gastrocnemius cross sectional area. Change is defined as the difference between baseline and endpoint values. Data are presented as mean ± SEM; * (*p* < 0.05, RMANOVA).

## Appendix A

**Table A1:** Baseline, midpoint, and endpoint values for blood measurements.

Measurement	Time Point	CTL	AT	D	DAT
25(OH)D, ng/mL	Baseline	24.8 ± 2.1	25.2 ± 1.4	26.6 ± 1.2	27.1 ± 1.6
	Midpoint	29.5 ± 3.2 ^A^	31.2 ± 2.7 *^A^	54.1 ± 3.0 *^B^	62.0 ± 4.5 *^B^
	End Study	31.0 ± 3.3 *^A^	32.6 ± 3.1 *^A^	63.7 ± 5.0 *^B^	72.1 ± 5.2 *^B^
VDBP, µg/ mL	Baseline	110 ± 5	115 ± 3	111 ± 4	115 ± 5
	Midpoint	109 ± 6	109 ± 7	109 ± 5	104 ± 10
	End Study	115 ± 6	110 ± 4	110 ± 4	108 ± 7
Ionized Ca, mg/dL	Baseline	5.01 ± 0.05	5.09 ± 0.03	5.09 ± 0.03	5.04 ± 0.05
	Midpoint	5.00 ± 0.03	5.12 ± 0.06	5.09 ± 0.04	4.97 ± 0.06
	End Study	4.98 ± 0.02	5.04 ± 0.04	5.09 ± 0.04	5.06 ± 0.07
Intact PTH, pg/ mL	Baseline	36.8 ± 3.6 ^A^	32.4 ± 2.6 ^A^	53.1 ± 5.5 ^B^	47.6 ± 5.0 ^B^
	Midpoint	43.1 ± 5.6	39.7 ± 3.8 *	39.9 ± 4.0 *	47.0 ± 6.3 *
	End Study	43.9 ± 6.9	37.8 ± 3.4 *	41.7 ± 3.5 *	41.5 ± 5.3 *
Insulin, µIU/mL	Baseline	15.3 ± 3.6	8.0 ± 1.1	9.22 ± 1.0	18.5 ± 6.3
	Midpoint	21.5 ± 9.7	12.2 ± 2.5	17.9 ± 3.6	26.9 ± 14.8
	End Study	10.7 ± 2.9	8.35 ± 1.2	11.1 ± 1.3	12.6 ± 3.0
Glucose, mg/dL	Baseline	99.0 ± 4.3	91.7 ± 4.2	97.2 ± 2.2	96.3 ± 1.5
	Midpoint	--	--	--	--
	End Study	97.6 ± 4.0	94.1 ± 2.1	98.2 ± 2.2	96.7 ± 2.8
HOMA-IR	Baseline	3.93 ± 1.04	1.78 ± 0.23	2.23 ± 0.25	4.34 ± 1.43
	Midpoint	--	--	--	--
	End Study	2.68 ± 1.02	1.95 ± 0.28	2.69 ± 0.32	3.02 ± 0.71

All values presented as mean ± SEM. * *p* < 0.05 vs baseline (paired samples *t*-test); values within a time point not sharing a letter are significantly different (*p* < 0.05, ANOVA); -- no data collected. AT, aerobic training; CTL, control; D, vitamin D; DAT, vitamin D + aerobic training.
